# Assessing the Impact of Pre-surgical Delay on Extradural Hematoma Outcomes

**DOI:** 10.7759/cureus.79250

**Published:** 2025-02-18

**Authors:** Zahid Khan, Seema Sharafat, Haidar Ali, Adnan Khan, Ahmad Noushad, Javaria Farman, Muhammad Sajjad

**Affiliations:** 1 Neurosurgery, Lady Reading Hospital Medical Teaching Institution, Peshawar, PAK; 2 Emergency and Trauma Unit, Lady Reading Hospital Medical Teaching Institution, Peshawar, PAK; 3 Pediatric Unit, Ali Medical Center, Peshawar, Peshawar, PAK; 4 Gynecology, Kalsoom Maternity Home, Peshawar, PAK; 5 Biological Sciences, Kohat University of Science and Technology, Kohat, PAK

**Keywords:** extradural hematoma, neurological outcomes, pre-surgical delay, recovery, survival, timeliness

## Abstract

Background: Extradural hematoma (EDH) is a life-threatening neurosurgical emergency, with timely surgical intervention critical to preventing neurological deterioration and improving patient outcomes. Delays in surgical treatment are a persistent concern, yet the specific impact of pre-surgical delays on clinical outcomes in EDH patients remains underexplored.

Objective: This study aimed to evaluate the impact of pre-surgical delay duration on clinical outcomes in patients with EDH, specifically examining survival rates, neurological status, and recovery outcomes.

Methodology: A retrospective observational study was conducted over a two-year period (January 2022 to December 2023). Patients aged 18 years and above, diagnosed with EDH and undergoing surgical intervention, were included. Data on pre-surgical delays, demographics, comorbidities, and clinical outcomes were collected from medical records. Statistical analyses included t-tests and multivariate regression to identify predictors of adverse outcomes.

Results: A total of 178 patients were analyzed. The mean pre-surgical delay was significantly longer in deceased patients (16.42 ± 6.24 hours) compared to survivors (7.92 ± 3.81 hours) (p < 0.001). Shorter pre-surgical delays (6.31 ± 2.52 hours) were associated with higher rates of full recovery compared to longer delays (12.18 ± 4.29 hours) (p < 0.001). Multivariate analysis identified pre-surgical delay, age, hypertension, and diabetes as significant predictors of adverse outcomes.

Conclusion: Pre-surgical delays significantly impact survival rates and recovery outcomes. Addressing logistical issues, resource constraints, and comorbid conditions is essential to minimize delays and improve patient prognosis.

## Introduction

Extradural hematoma (EDH) is a critical neurosurgical emergency that can result from head trauma, leading to the accumulation of blood between the dura mater and the inner skull [1]. EDH can exert significant pressure on the brain, resulting in severe neurological impairments or even death if left untreated [2]. Rapid diagnosis and timely surgical intervention are essential to mitigate the risk of complications and improve patient outcomes [3]. However, delays in surgical intervention remain a persistent concern, potentially exacerbating the severity of neurological damage [4].

The timely management of EDH has been well established as a key factor influencing patient prognosis, yet numerous factors contribute to delays in treatment [5]. These can include logistical constraints, challenges in early diagnosis, comorbidities, or resource limitations within healthcare settings [6]. While the relationship between surgical timing and outcomes has been explored in other conditions, the specific effects of pre-surgical delay on the outcomes of EDH require further investigation [7]. Understanding these delays’ impact on clinical results could provide insights into optimizing treatment pathways and improving healthcare systems [8].

Current evidence suggests that delays between diagnosis and intervention can lead to worsened neurological outcomes, including persistent morbidity and mortality [9]. These findings emphasize the urgency of timely intervention but also point to a need for a deeper exploration of how various factors related to delay may influence recovery trajectories [10]. Analyzing these aspects could lead to better-informed clinical decisions and strategies for intervention protocols, particularly in emergency neurosurgical care [11].

Despite existing literature on EDH and its management, there is limited research specifically examining the association between the timing of surgical intervention and the short- and long-term outcomes of patients with EDH. Thus, further investigation into how pre-surgical delays correlate with prognosis is essential to identify clearer patterns and clinical predictors.

Research objective

This study aimed to investigate the relationship between pre-surgical delay and clinical outcomes in patients with EDH, focusing on how the timing of surgical intervention impacts recovery and survival rates.

## Materials and methods

Study design and setting

This study employed a retrospective observational design conducted at the Department of Neurosurgery and Accident and Emergency, Lady Reading Hospital Medical Teaching Institution (MTI), Peshawar, over a duration of two years, from January 2022 to December 2023.

Inclusion and exclusion criteria

Patients eligible for inclusion in this study were those diagnosed with EDH during the study period who underwent surgical intervention for EDH, were above 18 years old, and had complete medical records available for analysis. Conversely, patients were excluded if they were managed non-operatively, had incomplete or missing medical records, or had coexisting intracranial pathologies that could confound the study outcomes.

Sample size

The sample size was determined using a convenience sampling technique, resulting in 178 patients included in the study. While convenience sampling may introduce selection bias, efforts were made to minimize its impact. All patients diagnosed with EDH and undergoing surgical intervention during the study period were systematically screened for eligibility. Based on predefined inclusion and exclusion criteria, a total of 178 eligible patients were finally included. Patient selection was conducted based on objective clinical criteria rather than discretionary inclusion, reducing the risk of selection bias. This sample size was sufficient to provide adequate statistical power for meaningful analysis, facilitating the identification of key predictors related to pre-surgical delays and enhancing the reliability of the findings.

Data collection

This study retrospectively collected data from patient medical records, radiological reports, and surgical notes. The data collection followed a structured approach, beginning with demographic variables such as age, gender, comorbidities, and trauma history to assess their potential influence on clinical outcomes.

Given the retrospective nature of the study, the exact time of trauma was not consistently documented. Therefore, pre-surgical delay was defined as the time from diagnosis to surgical intervention, focusing on in-hospital delays.

Hematoma characteristics, including size and location, were recorded based on radiological assessments (CT scan reports), which were documented in hospital records. Hematoma locations were categorized as frontal, temporal, parietal, occipital, and posterior fossa. While this study primarily examined the impact of pre-surgical delays, we acknowledge that hematoma location may influence outcomes due to factors such as space availability and brainstem compression.

Clinical outcomes, including neurological status, survival rates, and postoperative recovery, were systematically evaluated using data extracted from hospital records to determine the relationship between pre-surgical delays and patient prognosis.

The preoperative neurological status of all patients was assessed using the Glasgow Coma Scale (GCS) at the time of hospital admission. Postoperative GCS was recorded at discharge to evaluate neurological recovery and outcomes. Preoperative GCS scores were categorized as mild (13-15), moderate (9-12), or severe (≤8). Postoperative GCS was documented at discharge to assess neurological improvement and correlate with clinical outcomes.

Neurological recovery was categorized based on the patient’s postoperative functional status at discharge. "Fully recovered" was defined as the complete resolution of neurological deficits with a return to baseline functional capacity. "Improved" included patients who showed significant neurological recovery but had mild residual deficits not affecting daily activities. "Stable but with minor deficits" referred to patients with persistent neurological impairments requiring rehabilitation but not severely limiting function. "Poor recovery/no improvement" was categorized for patients who did not exhibit significant neurological improvement.

The assessment of return to normal activities was conducted at a follow-up visit three months postoperatively. Patients were categorized as "Returned to normal activities" if they resumed their pre-injury lifestyle without major restrictions. "Partially returned to normal" included those who could perform daily tasks with some limitations but required assistance or modifications. "Did not return to normal" was applied to patients who remained significantly impaired and unable to regain preoperative independence.

Finally, the study examined factors contributing to surgical delays, such as logistical issues, comorbidities, and resource constraints, while acknowledging that pre-hospital delays may also influence outcomes but were beyond the study’s scope.

The pre-surgical delay was categorized into three time intervals for clearer interpretation: less than six hours, six to 12 hours, and >12 hours from diagnosis to surgical intervention. These intervals were chosen based on clinical relevance and prior literature, allowing for a more structured assessment of delays and their impact on patient outcomes.

Statistical analysis

Data were analyzed using descriptive and inferential statistical methods. Continuous variables were expressed as mean ± standard deviation, while categorical variables were expressed as percentages. Pre-surgical delay categories (less than six hours, six to 12 hours, and >12 hours) were analyzed for their association with clinical outcomes. Associations between pre-surgical delays and patient outcomes were evaluated using t-tests as appropriate. Multivariate regression analysis identified predictors of adverse outcomes associated with delays in surgical intervention. A p-value of <0.05 was considered statistically significant. All analyses were conducted using the statistical software SPSS version 26 (IBM Corp., Armonk, NY).

Ethical approval

Ethical approval was obtained from the Institutional Review Board (Ref. No.: 106/LRH/MTI; dated: December 16, 2021) prior to initiating the study. Patient confidentiality was maintained by anonymizing all collected data. Informed consent was waived for this retrospective study, as per ethical guidelines and approval from the IRB.

## Results

Table 1 summarizes the demographic and trauma characteristics of the study population, consisting of 120 males (67.41%) and 58 females (32.59%), with mean ages of 44.8 ± 15.2 and 46.2 ± 16.3 years, respectively. Hypertension was more common in males (60.00%) compared to females (37.93%), while diabetes mellitus affected 33.33% of males and 20.68% of females. Smoking history was significantly higher in males (43.33%) than in females (6.89%). Road traffic accidents were the most common trauma cause, affecting 85.00% of males and 55.17% of females, followed by falls (46.67% males, 50.00% females) and assaults (16.67% males, 10.34% females).

**Table 1:** Demographic and trauma characteristics of the study population.

Variable		Male (n, %)	Female (n, %)
Age (years)	Mean ± SD	44.8 ± 15.2	46.2 ± 16.3
Gender	Male	120 (67.41%)	-
	Female	-	58 (32.59%)
Comorbidities	Hypertension	72 (60.00%)	22 (37.93%)
	Diabetes mellitus	40 (33.33%)	12 (20.68%)
	Smoking history	52 (43.33%)	4 (6.89%)
Trauma history	Road traffic accident	102 (85.00%)	32 (55.17%)
	Fall	56 (46.67%)	29 (50.00%)
	Assault	20 (16.67%)	6 (10.34%)

**Figure 1:**
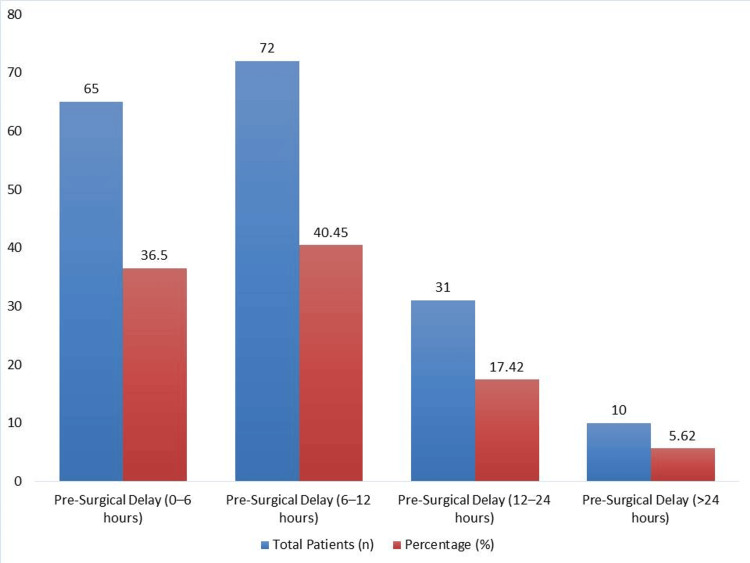
Distribution of patients based on pre-surgical delay intervals.

Figure 1 illustrates the distribution of patients based on pre-surgical delay intervals. The majority of patients (72, 40.45%) experienced a delay of six to 12 hours before surgery, followed by 65 patients (36.50%) who underwent surgery within zero to six hours. A smaller proportion of patients had delays of 12-24 hours (31, 17.42%), and only 10 patients (5.62%) experienced a delay exceeding 24 hours. This highlights that nearly three-quarters of patients underwent surgical intervention within the first 12 hours post diagnosis.

**Figure 2:**
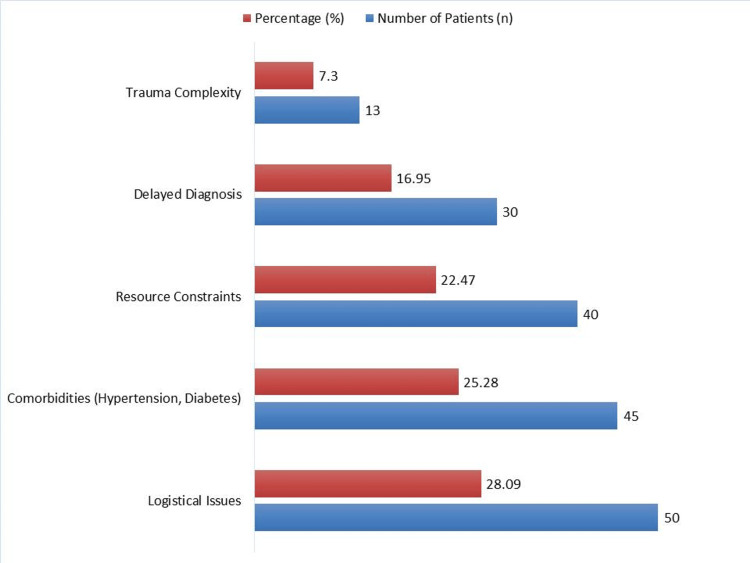
Key factors contributing to delays in surgical intervention.

Figure 2 highlights the key factors contributing to delays in surgical intervention among patients with EDH. Logistical issues, including patient transport delays, bed availability, and operating room scheduling, were the most common cause, affecting 50 patients (28.09%). Comorbidities such as hypertension, diabetes, and other pre-existing conditions contributed to delays in 45 patients (25.28%). Resource constraints, including limited surgical staff, ICU bed shortages, and equipment unavailability, were responsible for delays in 40 patients (22.47%). Delayed diagnosis, primarily due to atypical presentations, initial misdiagnosis, or delayed imaging, impacted 30 patients (16.95%). Trauma complexity, where multiple injuries required prioritization of other life-threatening conditions before EDH intervention, was the least frequent factor, affecting 13 patients (7.30%).

Table 2 presents the clinical outcomes of 178 patients following surgical intervention. Among them, 95.50% (170) survived, while 4.49% (8) deceased. Preoperatively, 40.45% (72) of patients had mild GCS scores (13-15), 36.50% (65) had moderate GCS scores (9-12), and 23.03% (41) had severe GCS scores (≤8). At discharge, neurological improvement was evident, with 69.66% (124) classified as mild, 21.34% (38) as moderate, and 8.99% (16) as severe based on GCS scores. Among survivors, 52.94% (90) fully recovered, 35.29% (60) showed improvement, and 11.76% (20) had minor neurological deficits, with no cases of poor recovery. Regarding functional recovery, 70.58% (120) returned to normal activities, 26.47% (45) partially returned, and 2.94% (5) did not regain normal function. Pre-surgical delays varied, with 61.80% (110) undergoing surgery within less than six hours, 25.28% (45) between six and 12 hours, and 12.92% (23) after >12 hours.

**Table 2:** Clinical outcomes of patients following surgical intervention. GCS: Glasgow Coma Scale.

Clinical outcome		Number of patients (n)	Percentage (%)
Preoperative GCS score	Mild (GCS = 13-15)	72	40.45%
	Moderate (GCS = 9-12)	65	36.50%
	Severe (GCS ≤ 8)	41	23.03%
Postoperative GCS score at discharge	Mild (GCS = 13-15)	124	69.66%
	Moderate (GCS = 9-12)	38	21.34%
	Severe (GCS ≤ 8)	16	8.99%
Survival status	Survived	170	95.50%
	Deceased	8	4.49%
Neurological status at discharge (surviving patients only)	Fully recovered	90	52.94%
	Improved	60	35.29%
	Stable but with minor deficits	20	11.76%
	Poor recovery/no improvement	0	0.00%
Recovery outcomes (surviving patients only)	Returned to normal activities	120	70.58%
	Partially returned to normal	45	26.47%
	Did not return to normal	5	2.94%
Pre-surgical delay	<6 hours	110	61.80%
	6-12 hours	45	25.28%
	>12 hours	23	12.92%
Follow-up at 3 months	Fully recovered at follow-up	135	79.41%
	Required further rehabilitation	35	20.59%

Table 3 presents the relationship between pre-surgical delay, hematoma characteristics, and patient outcomes. The mean pre-surgical delay was significantly shorter for survivors (7.92 ± 3.81 hours) compared to deceased patients (16.42 ± 6.24 hours, p < 0.001). Similarly, hematoma size was notably larger in deceased patients (45.27 ± 10.39 cm³) than in survivors (32.14 ± 8.51 cm³). Hematoma location also influenced survival, with frontal hematomas being more frequent among survivors (38.23%) and parietal or occipital hematomas more common in deceased patients (31.25% and 18.75%, respectively).

**Table 3:** Relationship between pre-surgical delay and patient outcomes.

Outcome		Pre-surgical delay (mean ± SD)	Hematoma size (mean ± SD, cm³)	Hematoma location (%)	p-value
Survival status	Survived	7.92 ± 3.81 hours	32.14 ± 8.51 cm³	Frontal (38.23%), temporal (29.41%), parietal (14.11%), occipital (9.41%), posterior fossa (8.82%)	<0.001*
	Deceased	16.42 ± 6.24 hours	45.27 ± 10.39 cm³	Frontal (12.50%), temporal (25.00%), parietal (31.25%), occipital (18.75%), posterior fossa (12.50%)	
Neurological status at discharge	Fully recovered	6.31 ± 2.52 hours	27.89 ± 6.82 cm³	Frontal (41.82%), temporal (26.36%), parietal (12.73%), occipital (11.82%), posterior fossa (7.27%)	<0.001*
	Improved	9.27 ± 3.63 hours	34.02 ± 7.14 cm³	Frontal (35.00%), temporal (30.00%), parietal (15.00%), occipital (10.00%), posterior fossa (10.00%)	
	Stable with minor deficits	12.18 ± 4.29 hours	40.13 ± 9.27 cm³	Frontal (21.43%), temporal (28.57%), parietal (21.43%), occipital (14.29%), posterior fossa (14.29%)	
Recovery outcomes	Returned to normal activities	6.86 ± 2.97 hours	30.41 ± 7.34 cm³	Frontal (40.83%), temporal (27.50%), parietal (13.33%), occipital (10.00%), posterior fossa (8.33%)	<0.001*
	Partially returned	10.33 ± 3.54 hours	38.92 ± 8.57 cm³	Frontal (25.00%), temporal (30.00%), parietal (20.00%), occipital (15.00%), posterior fossa (10.00%)	
	Did not return to normal	16.79 ± 5.82 hours	47.63 ± 11.20 cm³	Frontal (14.29%), temporal (28.57%), parietal (28.57%), occipital (14.29%), posterior fossa (14.29%)	

Neurological outcomes at discharge were closely linked to pre-surgical delays and hematoma characteristics. Fully recovered patients had the shortest pre-surgical delay (6.31 ± 2.52 hours) and the smallest hematoma size (27.89 ± 6.82 cm³, p < 0.001). Conversely, patients with minor neurological deficits had longer delays (12.18 ± 4.29 hours) and larger hematomas (40.13 ± 9.27 cm³). The distribution of hematoma location showed that frontal hematomas were more frequently associated with full recovery (41.82%), whereas posterior fossa hematomas were less common among those who fully recovered (7.27%).

Functional recovery followed a similar trend. Patients who returned to normal activities had shorter pre-surgical delays (6.86 ± 2.97 hours) and smaller hematomas (30.41 ± 7.34 cm³). In contrast, those who did not regain normal function had significantly longer delays (16.79 ± 5.82 hours) and larger hematomas (47.63 ± 11.20 cm³). Parietal and occipital hematomas were more common among patients with limited recovery, suggesting that hematoma location plays a critical role in determining post-surgical outcomes.

Table 4 presents the multivariate regression analysis of predictors for adverse outcomes. Pre-surgical delay remained a strong independent predictor, with an odds ratio of 1.16 (95% CI: 1.10-1.23, p < 0.001), indicating that each additional hour of delay increased the likelihood of adverse outcomes. Hematoma size also significantly influenced prognosis, with larger hematomas (per cm³ increase) associated with a 19% higher risk of adverse outcomes (OR = 1.19, 95% CI: 1.11-1.28, p < 0.001).

**Table 4:** Multivariate regression analysis of predictors for adverse outcomes. * P-value < 0.05 is significant.

Variable		Regression coefficient (β)	Odds ratio (OR)	95% CI for OR	p-value
Pre-surgical delay (hours)		0.15	1.16	1.10–1.23	<0.001*
Hematoma size (cm³)		0.08	1.19	1.11–1.28	<0.001*
Hematoma location	Frontal (ref)	—	—	—	—
	Temporal	0.25	1.28	1.01–1.63	0.045*
	Parietal	0.3	1.35	1.05–1.74	0.021*
	Occipital	0.35	1.42	1.05–1.92	0.023*
	Posterior fossa	0.4	1.49	1.10–2.02	0.015*
Age (years)		0.05	1.05	1.02–1.08	0.002*
Hypertension		0.4	1.49	1.10–2.02	0.015*
Diabetes mellitus		0.35	1.42	1.05–1.92	0.023*
Logistical issues		0.25	1.28	1.01–1.63	0.045*
Resource constraints		0.3	1.35	1.05–1.74	0.021*

Hematoma location was another key determinant. Compared to frontal hematomas, posterior fossa hematomas had the highest odds of adverse outcomes (OR = 1.49, 95% CI: 1.10-2.02, p = 0.015), followed by occipital (OR = 1.42, p = 0.023), parietal (OR = 1.35, p = 0.021), and temporal hematomas (OR = 1.28, p = 0.045). Other significant predictors included older age (OR = 1.05, p = 0.002), hypertension (OR = 1.49, p = 0.015), and diabetes mellitus (OR = 1.42, p = 0.023). Logistical issues and resource constraints also contributed to adverse outcomes (OR = 1.28 and OR = 1.35, respectively), underscoring the impact of systemic healthcare factors on patient survival and recovery. These findings highlight the critical role of timely surgical intervention in improving patient outcomes. Minimizing pre-surgical delays, managing comorbidities, and optimizing resource allocation could significantly enhance survival and neurological recovery among patients with EDH.

## Discussion

The findings of this study emphasize the significant impact of pre-surgical delay on the clinical outcomes of patients with EDH. Among the 178 patients analyzed, the mean pre-surgical delay was 7.92 ± 3.81 hours for survivors, whereas non-survivors experienced a substantially longer delay of 16.42 ± 6.24 hours (p < 0.001). These results align with previous research underscoring the critical importance of timely surgical intervention in improving neurological outcomes and reducing mortality in EDH patients [12].

Several studies have established precise thresholds for pre-surgical delays that significantly influence patient prognoses. Haque et al. [13] reported that delays beyond six hours are associated with a marked increase in morbidity and mortality among patients with traumatic brain injuries, including EDH. Sharif et al. [14] further categorized patients based on the time from trauma to surgical evacuation, demonstrating a 90% favorable outcome rate for those operated on within one hour, which dropped to 70% for delays of one to six hours and further declined to 50% for delays exceeding six hours. Wardak et al. [15] found that surgical intervention within the first hour post trauma yielded the best patient outcomes, whereas delays beyond this critical window were associated with significantly worse prognoses. Rahimi et al. [16] reported that every hour of delay beyond a certain threshold further increases the likelihood of unfavorable outcomes, reinforcing the urgent need for rapid diagnosis and surgical intervention in EDH management.

Our study was conducted at Lady Reading Hospital, one of the largest tertiary care centers in the region, which serves as a major referral center for traumatic brain injuries, including EDH. The high volume of EDH cases observed in this study can be attributed to the hospital’s role as a trauma center, receiving emergency referrals from surrounding districts and rural areas. The presence of a dedicated neurosurgical unit and emergency care infrastructure ensures that a large number of head trauma cases are treated here, providing a comprehensive dataset for analyzing the impact of surgical delays on outcomes.

The neurological status of patients at discharge also reflected the impact of surgical timing. Patients with shorter pre-surgical delays (6.31 ± 2.52 hours) exhibited significantly higher rates of full recovery compared to those with longer delays (12.18 ± 4.29 hours, p < 0.001). Additionally, preoperative GCS scores were found to be a key determinant of post-surgical outcomes. Patients presenting with a lower preoperative GCS had a higher likelihood of poor recovery, irrespective of surgical timing. This reinforces the importance of incorporating preoperative neurological status into outcome assessments, as a patient with a GCS of 3 may have a poor prognosis despite early intervention.

A critical point in evaluating delays is distinguishing between trauma-to-surgery and diagnosis-to-surgery time intervals. Due to the retrospective nature of our study, the exact time of trauma was not consistently documented in hospital records. Therefore, our analysis focused on in-hospital delays, defined as the time from diagnosis to surgical intervention. While this approach allows for a more structured assessment of healthcare system efficiency, it is acknowledged that trauma-to-surgery delays could provide additional insights into patient outcomes. Future prospective studies should aim to capture both time points for a more comprehensive analysis.

Resource constraints, including shortages of medical personnel, surgical equipment, ICU beds, and diagnostic tools, along with logistical challenges such as patient transportation delays, hospital workflow inefficiencies, emergency department overcrowding, and bureaucratic hurdles, were major contributors to surgical delays in this study. These factors affected 50% and 45% of patients, respectively. Such findings are consistent with previous research identifying limited healthcare resources, personnel shortages, and equipment unavailability as critical barriers to timely EDH management [17]. Additionally, comorbidities played a significant role, with hypertension affecting 60% of male patients and 37.93% of female patients. These results align with prior studies indicating that pre-existing medical conditions contribute to diagnostic and treatment delays, further increasing the risk of adverse outcomes in EDH patients [18].

Furthermore, the strong association between resource constraints and treatment delays underscores the need for systemic improvements in healthcare infrastructure and emergency management protocols. These findings reaffirm previous research highlighting healthcare resource limitations as key barriers to timely EDH management [19]. Additionally, the impact of comorbidities, such as hypertension and diabetes mellitus, on delayed diagnosis and adverse outcomes emphasizes the necessity of early screening and optimized perioperative care for at-risk patients [20].

In terms of recovery, patients who underwent surgery within six to 12 hours had significantly higher rates of returning to normal activities (70.58%) compared to those experiencing longer delays (16.79 ± 5.82 hours, p < 0.001). To ensure consistency in comparing recovery outcomes, follow-up assessments were conducted three months postoperatively. This standardized time frame allowed for a more accurate evaluation of functional recovery and minimized discrepancies in outcome reporting. The results reinforce previous findings emphasizing that reducing pre-surgical delays is essential for improving functional recovery and minimizing long-term disability [21].

Study strengths and limitations

One of the major strengths of this study is its large sample size, representing a high-volume trauma center’s experience in managing EDH patients. Conducted at MTI Lady Reading Hospital, Peshawar, a major referral center for neurosurgical emergencies, this study benefits from a diverse patient population across Khyber Pakhtunkhwa and other provinces. The availability of 24/7 neurosurgical services, advanced imaging, and a well-equipped emergency department allows for efficient triage and surgical intervention, making the findings applicable to similar trauma centers worldwide. The retrospective observational design allowed the use of readily available medical records, enhancing the feasibility of data collection. However, several limitations should be acknowledged. The use of convenience sampling may introduce selection bias, though efforts were made to systematically screen all eligible patients to minimize this effect. The retrospective nature of the study limits causal inferences. Although hematoma location was documented, statistical comparisons were not the primary focus of this study. Future prospective studies may further explore the role of hematoma location in determining clinical outcomes. Another key limitation is the reliance on medical records, which may lead to inaccuracies in data reporting. Importantly, this study primarily focuses on in-hospital delays (time from diagnosis to surgical intervention) due to inconsistent documentation of trauma onset. As a result, pre-hospital delays, including transportation time, initial triage, and emergency response efficiency, were not systematically analyzed, potentially underestimating the true impact of surgical delay on patient outcomes. Finally, the exclusion of non-operatively managed patients may limit the generalizability of findings to the broader EDH population.

## Conclusions

This study aimed to evaluate the impact of pre-surgical delay duration on clinical outcomes in patients with EDH, specifically examining survival rates, neurological status, and recovery outcomes. Our findings demonstrate that prolonged surgical delays are strongly associated with increased mortality, poorer neurological outcomes, and reduced chances of full recovery. Notably, non-survivors experienced significantly longer pre-surgical delays (16.42 ± 6.24 hours) compared to survivors (7.92 ± 3.81 hours, p < 0.001). Patients who underwent surgery within six to 12 hours had a higher likelihood of returning to normal activities (70.58%) than those with longer delays. Moreover, hematoma size and location played a crucial role in patient outcomes, with space-occupying lesions in critical regions (such as the posterior fossa) leading to worse prognoses. However, due to the retrospective nature of the study, establishing a direct causal relationship between surgical delay and outcomes remains challenging.

Multivariate analysis identified pre-surgical delay, advanced age, preoperative mental status (assessed using the GCS), and comorbid conditions such as hypertension and diabetes as significant predictors of adverse outcomes. Additionally, postoperative imaging findings were assessed to evaluate recovery and complications, which further emphasized the impact of surgical timing. These results highlight the urgent need for timely surgical intervention to improve patient prognosis and minimize complications associated with EDH. Optimizing hospital protocols, enhancing emergency response systems, streamlining hospital workflows, and addressing resource constraints are critical for reducing surgical delays. Strengthening healthcare infrastructure and trauma care systems will be pivotal in improving survival rates and functional recovery in EDH patients.
